# Interaction of pregnane X receptor with hypoxia-inducible factor-1 regulates chemoresistance of prostate cancer cells

**DOI:** 10.20517/cdr.2023.14

**Published:** 2023-06-16

**Authors:** Jiuhui Wang, Daotai Nie

**Affiliations:** ^1^Department of Medical Microbiology, Immunology and Cell Biology, Southern Illinois University School of Medicine, Springfield, IL 62794, USA.; ^2^Simmons Cancer Institute, Southern Illinois University School of Medicine, Springfield, IL 62794, USA.

**Keywords:** PXR, HIF-1, prostate cancer, chemoresistance, multidrug resistance, MDR1, hypoxia

## Abstract

**Aim:** The nuclear pregnane X receptor (PXR) is a pivotal regulator of steroid and xenobiotics metabolism and plays an important role in shaping tumor cell responses to chemotherapy. Hypoxia within tumor tissue has multifaceted effects, including multiple drug resistance. The goal of this study was to determine whether PXR contributes to hypoxia-induced drug resistance.

**Methods:** Metastatic prostate cancer cells were used to study the interaction of PXR and hypoxia-inducible factor-1 (HIF-1 in drug resistance associated with hypoxia. The activities of PXR and HIF-1 were determined by assays for its reporter gene or target gene expression. Co-immunoprecipitation (Co-IP) was used to determine the interaction of PXR and HIF-1. Ablation or inhibition of PXR or HIF-1 was used to determine their roles in hypoxia-induced chemoresistance.

**Results:** PXR was activated by hypoxia, leading to increased expression of multidrug resistance protein 1 (MDR1). Inhibition of PXR by pharmacological compounds or depletion by shRNAs reduced the hypoxic induction of MDR1 and sensitized prostate cancer cells to chemotherapy under hypoxia. HIF-1 was required for PXR activation under hypoxia. Co-immunoprecipitation results showed that HIF-1 and PXR could physically interact with each other, leading to crosstalk between these two transcription factors.

**Conclusion:** PXR contributes to hypoxia-induced drug resistance in prostate cancer cells through its interaction with HIF-1.

## INTRODUCTION

Hypoxia is a common occurrence in solid tumors as a result of the limited vasculature and the deregulated proliferation of cancer cells^[1]^. Tumor hypoxia elicits profound changes in cellular behaviors that influence cellular metabolism, genetic stability, angiogenesis, and self-renewal^[2,3]^. Tumor cells in the hypoxic regions are more resistant to chemotherapy than the cells in normoxic regions^[4]^. Elucidation of the mechanism involved in hypoxia-induced drug resistance can lead to new approaches to improve the efficacy of chemotherapy.

Hypoxia-induced factors (HIFs), which belong to the basic-helix-loop-helix family of transcription factors, play a central role in cellular and systemic responses to oxygen deficiency^[5]^. HIF-1, the hypoxia-inducible subunit of heterodimeric HIF-1, has been suggested as a promoter of tumorigenesis. The association between HIF-1 overexpression, treatment failure, and poor prognosis has been extensively reported^[6-8]^. HIF-1 plays an important role in hypoxia-induced drug resistance and could be an interventional target for drug resistance^[9,10]^. While the protective effects of HIF-1 against chemotherapy are attributed to its interference with cell cycle arrest and apoptosis and induction of multidrug resistant gene (MDR1) expression^[11,12]^, the molecular mechanisms underlying the HIF-1-mediated chemoresistance remain elusive.

Pregnane X receptor (PXR, NR1I2), a member of the nuclear receptor superfamily, is also known as steroid and xenobiotic receptor. PXR regulates cellular response to xenobiotics through the induction of drug-metabolic enzymes (DME) and transporters. Upon binding with ligands and activation, PXR translocates from the cytoplasm to the nucleus and regulates the transcription of target genes^[13]^. The significance of PXR in cancer chemoresistance is underlined by its activation by commonly used agents of chemotherapy, and its putative role in the regulation of the expression of DMEs and efflux transporters. We have reported that activation of PXR with agonists led to increased resistance of prostate and breast cancer cells to Taxol, vinblastine and tamoxifen, indicating an important role of PXR in chemoresistance^[14,15]^.

Prostate cancer is one of the most common malignancies affecting men, and chemotherapy is part of the standard of care for metastatic castration-resistant prostate cancer. In this study, we tested the hypothesis that PXR is a determinant of hypoxia-induced drug resistance in prostate cancer. Here, we report that HIF-1 activated PXR under hypoxic conditions, leading to increased expression of MDR1 and increased resistance of prostate cancer cells towards chemotherapy. Interestingly, activated PXR and HIF-1 physically interacted with each other. These results suggest an intricate interaction between PXR and HIF-1 in shaping the hypoxic tumor response to chemotherapy.

## METHODS

### Materials

Phoenix Ampho and 293T packaging cell lines were purchased from Allele Biotechnology. The human prostate cancer cell lines LNCaP, DU145 and PC-3 were obtained from American Type Culture Collection. PXR (G-1) mouse monoclonal antibodies were purchased from Santa Cruz Biotechnology. HIF-1 rabbit monoclonal antibodies were purchased from Cell Signaling Technology. Myc-tag mouse monoclonal antibodies were purchased from Applied Biological Materials Inc. The real-time polymerase chain reaction (PCR) reagents, dual luciferase reporter assay system, and 3-(4,5-dimethylthiazol-2-yl)-5-(3-carboxymethoxyphenyl)-2-(4-sulfophenyl)-2H-tetrazolium (MTS)-based cell viability assay kits were obtained from Promega Corporation. DNAfectin liposome transfection reagents were purchased from Applied Biological Materials Inc. Apoptosis Assay kit was purchased from R & D Systems. Rifampicin was purchased from Tocris Bioscience. Ketoconazole, puromycin, and doxorubicin were obtained from Sigma. Taxol was obtained from Enzo Life Science. Doxycycline and cobalt chloride were obtained from Fisher Scientific. pGL3-HRE and pDNA3-HIF-1 were purchased from Addgene Company. PCDH (pCDH vector, purchased from System Bioscience.)-myc-HIF-1, pGL3-PXRE, and pBabe-PXR were constructed in our lab. The pGIPZ lentiviral shRNA constructs against HIF-1 and pTRIPZ lentiviral shRNA constructs against human PXR were purchased from Open Biosystem.

### Cell culture and stable transfection

Prostate cancer cells were grown in Roswell Park Memorial Institute (RPMI) 1,640 medium with 10% Fetal bovine serum (FBS) and 1% antibiotics under an atmosphere containing 5% CO_2_ at 37 °C in a humidified incubator. Phoenix Ampho cells were grown in Dulbecco’s Modified Eagle Medium (DMEM) with 10% FBS and 1% antibiotics. Regarding HIF-1 pGIPZ lentiviral shRNA and PXR pTRIPZ lentiviral shRNA viral preparations, 293T cells were transfected with the shRNA constructs in the presence of packaging plasmids (System Bioscience), according to the manufacturer’s protocols. LNCaP cells with HIF-1 GIPZ lentiviral shRNA expression were marked by green florescent protein (GFP) and selected by fluorescence-activated cell sorting (FACS). LNCaP cells with PXR pTRIPZ lentiviral shRNA expression were regulated by a Tet-On system and marked by red fluorescent protein (RFP) in the presence of doxycycline treatment. For cell culture under hypoxia, hypoxia was induced by GasPak EZ Anaerobe Pouch System (Becton Dickinson and Company).

### Immunocytochemistry

LNCaP cells were seeded into six-well plates containing cover glass and incubated under normoxic or hypoxic conditions for 24 h. Cells were fixed with 2% paraformaldehyde for 15 min, permeabilized with 0.1% Triton X-100 for 1 min, and blocked with 1% bovine serum albumin in PBS for 30 min. Cells were then incubated overnight at 4 °C with mouse monoclonal anti-PXR primary antibody at 1:100 dilution and sequentially incubated with Alexa Fluor 488 goat anti-mouse secondary antibody for 1 h at room temperature. The slides were then washed, mounted in Prolong Gold antifade reagent with 4’,6-diamidino-2-phenylindole (Molecular Probe), and visualized with a BX41 system microscope (Olympus).

### Cell viability assays

Cell viability and proliferation were MTS assay. Briefly, 5 × 10^4^ cells were seeded onto 96-well plates with 200 µL medium per well and incubated in standard cell culture conditions. After 24 to 72 h of incubation, 20 µL MTS was added to each well and incubated at 37 °C for 2 h. The OD values at 490 nm wavelength were then obtained.

### Anticancer drugs

To determine the sensitivity of tumor cell lines to anticancer drugs, the viabilities of cells after treatments were determined by MTS viability assay according to the manufacturer’s instructions. Specifically, under normoxic or hypoxic conditions, 1 × 10^4^ cells were incubated with different doses of anticancer drugs for 48 h. Hypoxia was induced by GasPak EZ Anaerobe Pouch System. After the treatments, the cells were incubated with the MTS solutions and the absorbances were measured at 490 nm.

Induction of apoptosis in tumor cells by Taxol was determined by two-color analysis using propidium iodide (PI) and FITC-conjugated annexin V based on the manufacturer’s instructions. After incubations with or without Taxol for 48  h under normoxic or hypoxic conditions, the cells were stained with PI and FITC-conjugated annexin V and analyzed with a flow cytometer.

### Real-time PCR

Total RNA was isolated from the cultured cells using the PerfectPure RNA Purification System (5 Prime). The quantity and quality of different RNA samples were determined by the 260:280 nm absorbance ratios. The RNA samples were reverse transcribed with random hexanucleotide primers and ProtoScript Moloney Murine Leukemia Virus (M-MuLV) Taq RT-PCR kit (New England Biolabs). The cDNA samples were used by real-time PCR using an Applied Biosystems 7,500 real-time PCR system. Sequences for the primers are listed in Table 1. The relative expression was calculated using the ΔΔCt method and normalized to β-actin RNA levels.

**Table 1:** Primers used in real-time PCR

**Gene**	**Forward primer**	**Reverse primer**
*HIF-1*	GCCACCACTACCACTGCCACC	GCTCTGTTTGGTGAGGCTGTCCG
*HIF-2*	AAGCATCCCTGCCACCGTGC	CAACGGCGCTGCTCCCAAGA
*HK1*	TGAAGGGCGGATCACCCCGG	CTGCTCGGCCAAGCGGTAGG
*HK2*	CCACGCGCCTGTGAATCGGAG	CTCATCAGAGAGGCGCATGTGGT
*PFKL*	AAGTGATGGGCCGGCACTGC	GCCGCACTGACTGGTTCCCC
*PKM2*	CTACCGGCCCGTTGCTGTGG	TTGCTGCCCAAGGAGCCACC
*PXR*	GCAGGAGCAATTCGCCATT	TCGGTGAGCATAGCCATGATC
*C-met*	CCAATGGCCTGCAGCCGTGA	TGCCGCTCCTGTCCTGAGCA
*EPO1*	GCTCACTCGGCACCCTGCAAA	TGCCACCAAGGGAGTGCCCA
*IGFβ*	GAGGGTGGAGCCTCCTGGGG	GCCTCCGAGCACCCTCCTGA
*LDHA*	TGCCACCTCTGACGCACCAC	GGCATGTTCAGTGAAGGAGCCAGG
*BNip3*	GGGGTGGCCACGTCACTTGT	AGTAGGTGCCTTCAGCAGAAAACTG
*NIX*	CCCAGATTTGTGTTGAACGA	ACGGGAACTTGTTGCACTTT
*β-actin*	GGACTTCGAGCAAGAGATGG	AGCACTGTGTTGGCGTACAG
*MDR1*	GGTTCAGGTGGCTCTGGATA	TGACTCCATCATCGAAACCA
*CYP3A4*	TGGCACCGTAAGTGGAGCCTGA	TGCAGTCCATTGGATGAAGCCCA

### Western blot

Cells were scraped off the plate and directly lysed with 2 × Sodium dodecyl-sulfate polyacrylamide gel electrophoresis (SDS-PAGE) electrophoresis sample buffer. The lysate was boiled, sonicated, and centrifuged. Supernatants were loaded into a 10% SDS-PAGE gel and transferred to a polyvinylidene difluoride PVDF membrane. The membrane was blocked for 1 hour and incubated with primary antibody overnight at 4 °C. After washing with Tris-buffered saline with 0.1% Tween® 20 detergent (TBS-T) three times for a total of 15 min, the membrane was incubated with fluorescently-labeled secondary antibody for 1 hour. After washing three times, the blots were scanned by an Odyssey infrared imaging system (LI-COR Biosciences).

### Luciferase assay

Cells were seeded onto 12-well plates and transfected with luciferase reporter constructs pGL3-PXRE or vector controls in the presence of the control Renilla luciferase construct. The pGL3-PXRE was the PXR reporter construct containing two PXR-responsive fragments in *CYP3A4* promoters as previously described^[14,15]^. Cells were harvested with the passive lysis buffer 48 h after transfection, and luciferase activity was determined by dual luciferase reporter assay system according to the manufacturer’s protocol (Promega).

### Co-immunoprecipitation assay

The 293T cells were transfected with pBabe-PXR or co-transfected with PCDH-myc-HIF-1 and pBabe-PXR. The transfected cells were lysed in a non-denaturing Radioimmunoprecipitation assay (RIPA) buffer (pH of 8.0) containing 20 mM Tris-HCl, 137 mM NaCl, 2 mM Ethylenediaminetetraacetic acid (EDTA), and 1% NP-40 containing protease inhibitors. The cellular lysates were agitated slowly at 4 °C for 20 min and clarified by centrifugation at 12,000 g at 4 °C for 20 min. To immunoprecipitate PXR, 1 mg supernatants were incubated overnight at 4 °C with 4 g mouse monoclonal anti-PXR antibody, followed by mixing of the cell lysates with 100 L protein G-coupled sepharose beads and agitation for 4 h at 4 °C. After washing 3 times with non-denaturing RIPA buffer, the beads were collected by centrifuging at 5,000 g at 4 °C for 1 min. The bound proteins were solubilized with SDS sample buffer and subjected to SDS-PAGE for Western Blot analyses.

### Statistics

Two-tailed Student’s t-tests with a significance level of 0.05 were used to analyze the differences between the two experimental groups. All results were expressed as mean ± SD.

## RESULTS

### PXR is activated by hypoxia and stimulates MDR1 expression

**Figure 1:**
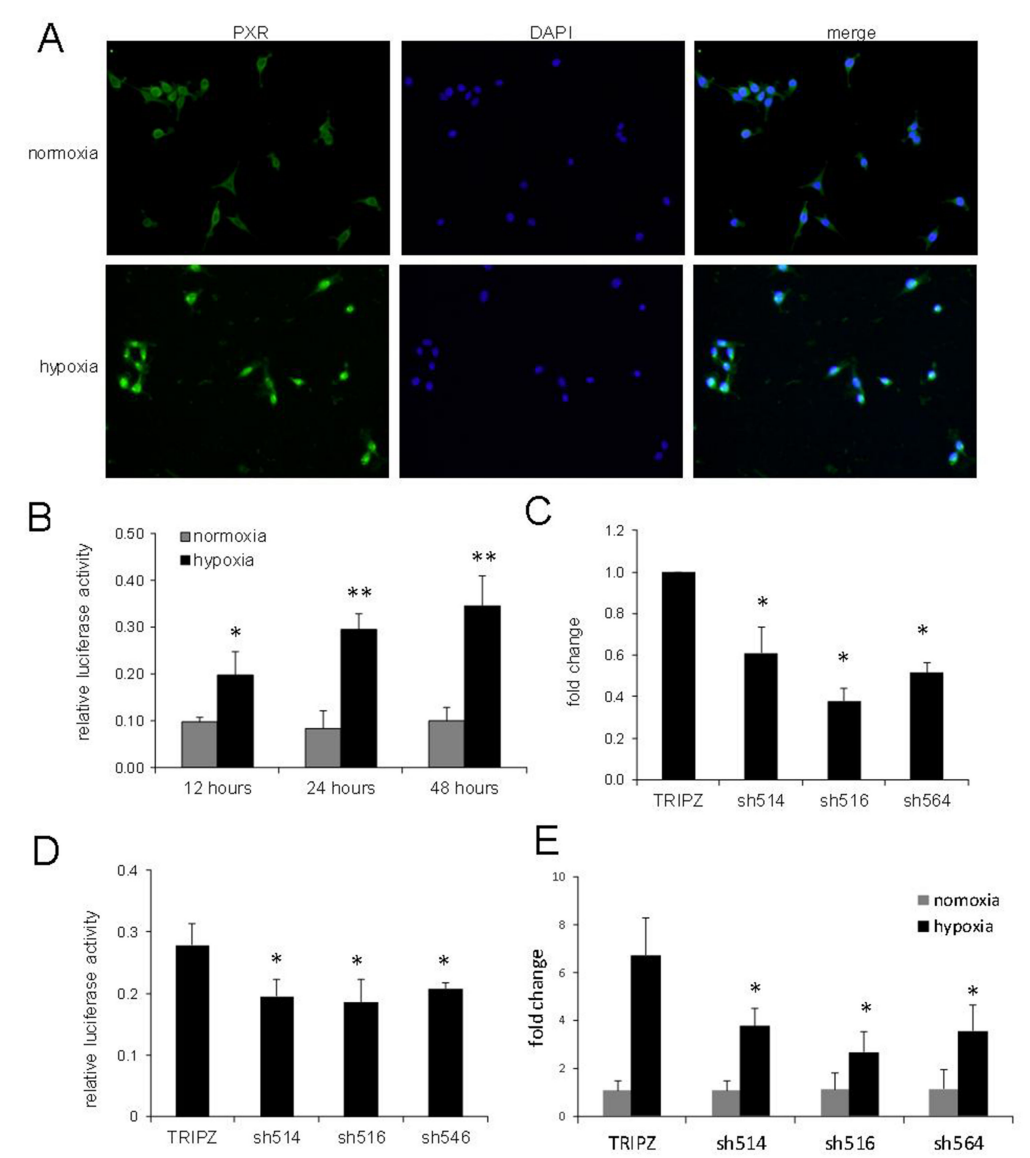
Activation of PXR by hypoxia. (A) Increased nuclear localization of PXR under hypoxia. LNCaP cells cultured under hypoxic or normoxic conditions for 24 h were processed for evaluation of PXR with mouse anti-PXR antibody and DAPI nuclear staining; (B) The activities of PXR in LNCaP cells cultured under normoxic and hypoxic conditions for the indicated durations, as measured by luciferase gene reporter assays; (C) The mRNA expression levels of PXR in LNCaP cell lines with stable expression shRNAs against PXR, as evaluated with real-time PCR; (D) PXR activities in LNCaP cells with stable expression shRNAs against PXR in hypoxia, as determined by luciferase assays; (E) Effects of hypoxia on mRNA expression of MDR1 in LNCaP cells with PXR expression knocked down, normalized with the values obtained from normoxic conditions. The results shown were from at least three independent experiments. Error bars represent standard deviation. *P < 0.05; **P < 0.01, when compared to normoxic controls (B) or vector TRIPZ controls (C, D, E). MDR1: multidrug resistance protein 1; PCR: polymerase chain reaction; PXR: pregnane X receptor. DAPI: 4’,6-diamidino-2-phenylindole; PCR: polymerase chain reaction.

Previously, we reported that PXR activation was responsible for increased resistance towards chemotherapy in prostate and breast cancers^[14,15]^. As a receptor for xenobiotics, PXR activation requires its translocation from the cytosol to the nucleus to regulate the expression of its target genes^[16]^. First, we performed immunofluorescence analysis and found that PXR was localized to the nucleus when cultured under hypoxic conditions but was mostly localized to the cytosol under normoxic conditions [Figure 1A]. Next, we determined whether hypoxia can affect the transcriptional activity of PXR using luciferase reporter gene assay. As shown in Figure 1B, compared to normoxic conditions, the activity of PXR was significantly increased in LNCaP cells when cultured under hypoxic conditions. The results indicated that PXR was activated and translocated from the cytosol to the nucleus under hypoxic conditions in LNCaP cells.

PXR activation can stimulate the expression of DMEs and efflux transporters, such as MDR1(*ABCB1*)^[14]^. To determine whether PXR activation by hypoxia can lead to altered expression of MDR1, we knocked down PXR expression in LNCaP cells using three independent shRNAs in LNCaP. As shown in Figure 1C and D, the shRNAs effectively suppressed mRNA expression and decreased the activity of PXR under hypoxic conditions. We then evaluated mRNA levels of MDR1 in LNCaP cells with PXR knockdown. In vector control cells, MDR1 expression had a 6.7-fold increase when cultured under hypoxic conditions compared to normoxic conditions [Figure 1E]. However, the hypoxia-stimulated 6.7-fold increase in MDR1 expression was reduced to only 2.7 to 3.8-fold in cells with PXR knocked down [Figure 1E], suggesting that PXR was at least partially responsible for MDR1 induction by hypoxia.

### PXR activation contributes to hypoxia-induced chemoresistance

**Figure 2:**
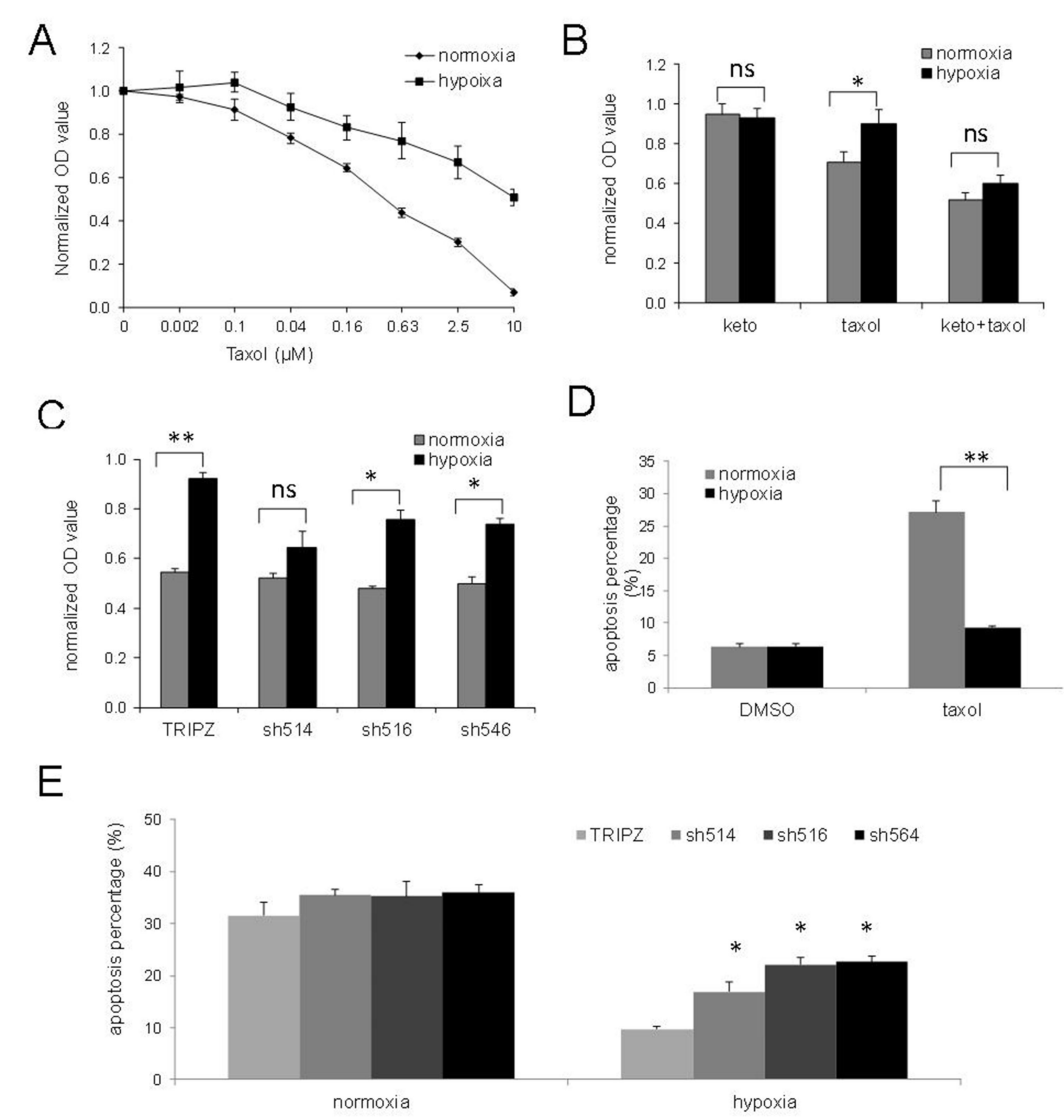
PXR’s role in hypoxia-induced chemoresistance. (A) Hypoxia increased resistance of LNCaP cells to Taxol at different dosages; (B) Effects of ketoconazole (10 M) on the sensitivity of LNCaP cells to Taxol (1 µM) under normoxic or hypoxic conditions; (C) Sensitivity of LNCaP cells with PXR knockdown to Taxol (1 M); (D) Hypoxia reduced apoptosis induced by Taxol; (E) Sensitization of hypoxic LNCaP cells toward Taxol-induced apoptosis. LNCaP cells with PXR knockdown treated with Taxol 1 µM were incubated in hypoxic condition for 48 h, and the percentage of apoptosis was determined by PI and annexin V staining followed by flow cytometry assay. The results shown were from at least three independent experiments. Error bars represent standard deviation. *P < 0.05. **P < 0.01. ns, not significant. PI: propidium iodide; PXR: pregnane X receptor.

To model the effects of hypoxia on the drug sensitivity of prostate cancer cells, we plated out and cultured LNCaP cells in media with 10% FBS, subjected them to normoxic or hypoxic conditions, and determined their survival by MTS assays after treatment with Taxol for 48 h. LNCaP cells were more resistant to Taxol under hypoxic conditions than those under normoxic conditions [Figure 2A]. Next, we determined whether PXR activation is responsible for chemotherapy resistance under hypoxic conditions. We evaluated whether hypoxia-induced chemoresistance can be reduced by ketoconazole, an antagonist of PXR which suppresses PXR activation via disruption of the interaction of PXR with the co-activator steroid receptor co-activator-1^[17]^. Results showed that treatment with ketoconazole restored the sensitivity of LNCaP cells to Taxol in hypoxic conditions [Figure 2B]. We further evaluated the effects of PXR knockdown on hypoxia-induced drug resistance. After treatment with Taxol in hypoxic conditions, the cell viability of these PXR knockdown cells was evaluated by MTS assays. Consistent with the results of antagonist experiments, knockdown of PXR by shRNAs restored the sensitivity to Taxol in one group (sh514 group) [Figure 2C]. However, in the other two groups (sh516 and sh546 groups), PXR knockdown reduced, but did not abolish, the hypoxia-induced drug resistance [Figure 2C].

Since cell viability can be multifactorial, we evaluated the apoptosis induced by Taxol via Annexin V staining in LNCaP cell lines under normoxic and hypoxic conditions. As shown in Figure 2D, the percentage of Taxol-induced apoptosis was reduced from 27% under normoxic conditions to 9% under hypoxic conditions. Taxol-induced apoptosis was also evaluated in PXR knockdown cells by Annexin V staining. Compared with the vector control, shRNA knockdown of PXR significantly increased the percentage of apoptotic cells under hypoxic conditions [Figure 2E]. These results suggest that PXR contributes to the increased resistance of hypoxic tumor cells to Taxol-induced apoptosis.

### HIF-1 is required for PXR activation under hypoxic conditions

**Figure 3:**
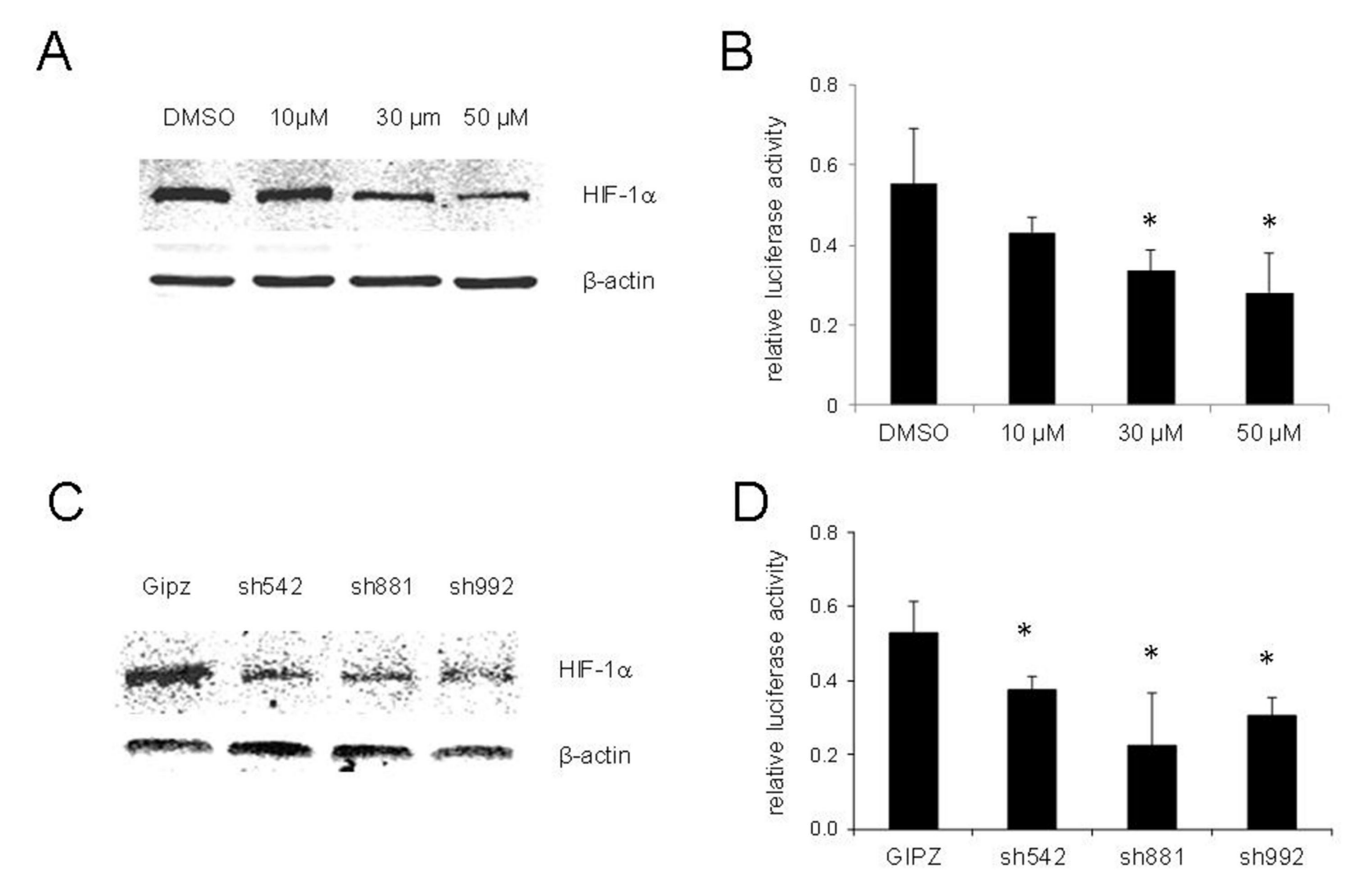
Inhibition or knockdown of HIF-1 suppressed the activity of PXR in hypoxia of LNCaP cells. (A) Expression of HIF-1 in LNCaP cells treatment with chrysin at the indicated concentrations and cultured under hypoxic conditions for 24 h. HIF-1 was detected by Western blot; (B) PXR activity of LNCaP cells treatment with chrysin at the indicated concentrations and cultured under hypoxic conditions for 24 h. PXR activity was determined by luciferase assay; (C) Expression of HIF-1 in LNCaP cells with stable expression shRNA against HIF-1 under hypoxic conditions. HIF-1 was detected by Western blot; (D) PXR activity of LNCaP cell lines with stable expression shRNA against HIF-1 under hypoxic conditions. The fold change was hypoxia/normoxia. The results shown are from three independent experiments. Error bars represent standard deviation. *P < 0.05. HIF-1: hypoxia-inducible factor-1; PXR: pregnane X receptor.

HIF-1 is a master regulator of adaptive responses to hypoxia and has been demonstrated to be related to hypoxia-induced chemoresistance. To test whether HIF-1 induction is required for PXR activation under hypoxic conditions, we determined the activities of PXR in LNCaP cells treated with chrysin, a natural flavonoid that reduces the stability and inhibits protein synthesis of HIF-1^[18]^. As shown in Figure 3A, chrysin suppressed the induction of HIF-1 under hypoxic conditions in a dose-dependent manner. Concurrently, the activity of PXR was significantly reduced [Figure 3B].

We also knocked down HIF-1 expression in LNCaP cells with three different shRNAs against HIF-1. The induction of HIF-1 under hypoxic conditions was significantly suppressed in shRNA-expressed cells [Figure 3C]. The activity of PXR in HIF-1 knockdown cell lines was examined, and the results shown in Figure 3D suggest that PXR activity was significantly decreased in cells with HIF-1 knockdown compared with the control cell line.

### Activation of PXR by HIF-1 and their interactions

**Figure 4:**
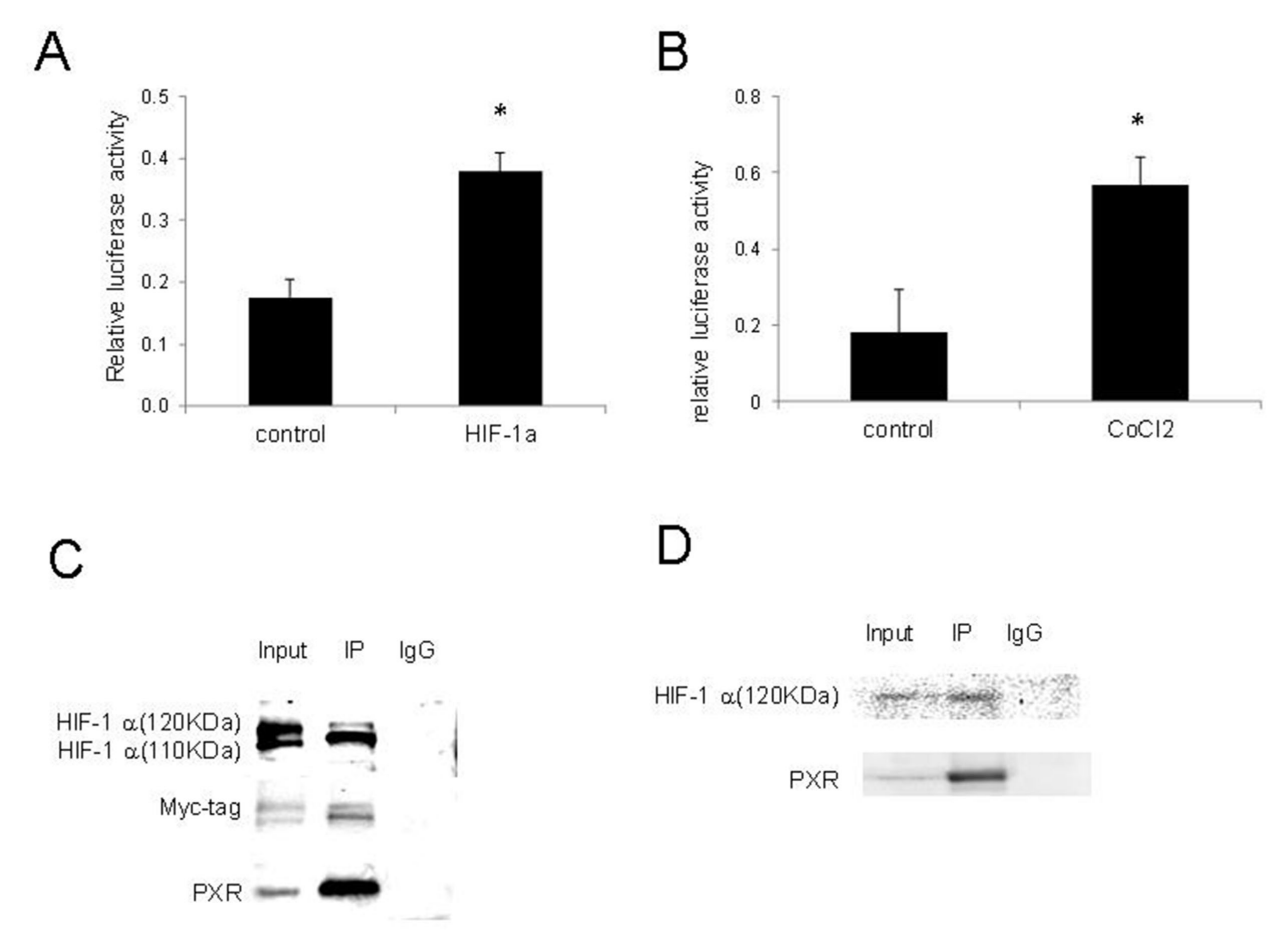
Activation of PXR by HIF-1 and their interactions. (A) HIF-1 increased PXR activity. 293T cells transiently co-transfected with pGL3-PXRE, PCDH-HIF-1, and pGL3-Renilla. PXR activity was determined by luciferase assay 48 h after transfection. The results shown are from three independent experiments. Error bars represent standard deviation. *Indicates significance (*P* < 0.05); (B) CoCl_2_ increased PXR activity. 293T cells transiently co-transfected with pGL3-PXR and pGL3-Renilla and incubated with CoCl_2_ 100 μM for 24 h. PXR activity was measured by luciferase assay; (C) Co-immunoprecipitation of overexpressed PXR and HIF-1. IP was performed with mouse anti-PXR, followed by immunoblotting with rabbit anti-HIF-1, rabbit anti-myc-tag, and mouse anti-PXR; (D) Co-IP between PXR with endogenous HIF-1 under hypoxic conditions. IP was performed with mouse anti-PXR, followed by immunoblotting with rabbit anti-HIF-1 and mouse anti-PXR. Co-IP: Co-immunoprecipitation; HIF-1: hypoxia-inducible factor-1; PCDH: pCDH vector; PXR: pregnane X receptor; pGL3-PXRE: PXR responsive element in PGL3 vector.

Since the PXR activation by hypoxia was compromised by HIF-1 inhibition by chrysin or knockdown by shRNAs, we determined whether HIF-1 can directly activate PXR. We co-transfected 293T cells with pDNA3-HIF-1 and pGPL2-PXRE and determined the effects of HIF-1 on PXR activities. The results shown in Figure 4A indicate that HIF-1 significantly increased the transcriptional activity of PXR. We also examined the changes in PXR activity in 293T cells following the induction of HIF-1 with CoCl_2_, a hypoxia-mimicking agent that stabilizes HIF-1^[19]^, and demonstrated that CoCl_2_ also significantly increased the activity of PXR [Figure 4B].

The finding that PXR could be activated by HIF-1 led us to investigate whether there was direct interaction between these two transcriptional factors. The lysate of 293T cells co-transfected with PCDH-myc-HIF1 and pBabe-PXR was subjected to immunoprecipitation with anti-PXR antibody and subsequently to Western blot with anti-HIF-1, anti-myc tag, and anti-PXR antibody. Two specific bands with molecular weights of about 120 KDa and 110 KDa were detected [Figure 4C], indicating a protein-protein interaction between ectopically expressed HIF-1 and PXR under normoxic conditions.

We further confirmed the interaction of PXR with endogenous HIF-1. After transfection with pBabe-PXR, 293T cells were cultured under hypoxic conditions for 24 h, and co-immunoprecipitation was performed as described in the Methods. We found that PXR bound to endogenous HIF-1 (120KDa) isoform under hypoxia conditions [Figure 4D]. These results suggest a protein-protein interaction between PXR and HIF-1 under hypoxic conditions.

## DISCUSSION

PXR has been reported to interact with other transcriptional factors including CAR, FXR, NF-B, and PPAR, suggesting its potential role in various non-canonical functions in addition to its role in metabolism of xenobiotics^[20-22]^. In the present study, we described the interaction and crosstalk between the nuclear receptor PXR and HIF-1, an important transcriptional factor that shapes cellular responses to changes in the availability of oxygen. Several lines of evidence suggest that the activation of PXR under hypoxic conditions was attributed to HIF-1: (1) forced or induced expression of HIF-1 increased PXR activity; (2) hypoxia-induced translocation of PXR from the cytoplasm to the nucleus; (3) HIF-1 inhibition by pharmacologic inhibition or shRNA knockdown abolished the activation of PXR under hypoxic conditions; (4) Co-IP analysis revealed a direct interaction between HIF-1 and PXR. These results strongly suggest that PXR activation by HIF-1 is one of the critical mechanisms underlying hypoxia-induced chemoresistance in prostate cancer cells.

Hypoxia-induced drug resistance poses challenges to effective cancer chemotherapy. Here, we confirmed that hypoxia caused drug resistance in prostate cancer cells. PXR is usually activated in response to xenobiotics, as well as many therapeutics, and plays a potential role in drug resistance during cancer treatment. In our previous study, we found that PXR was expressed in prostate cancer, and its activation led to the expression of MDR1 and *CYP3A4* and increased resistance to chemotherapeutics^[14,15]^. In this study, we found that hypoxia increased PXR activity and nuclear translocation of PXR in LNCaP cells. PXR knockdown with shRNA or inhibition of PXR activation with the antagonist ketoconazole sensitized tumor cells to Taxol under hypoxic conditions. These results suggest a critical role of PXR in hypoxia-induced chemoresistance in prostate cancer.

While hypoxia-induced drug resistance can be multifactorial, efflux transporters such as MDR1 can contribute to tumor cell resistance to chemotherapy. MDR1 is an ATP-dependent efflux pump with broad substrate specificity. By maintaining reduced intracellular concentrations of antitumor drugs, MDR1 confers cancer cells with multidrug resistance phenotype. Consistent with a previous study^[11]^, MDR1 expression was induced at the transcriptional level under hypoxic conditions. The induction of MDR1 by hypoxia was compromised by either PXR or HIF-1 knockdown, suggesting that PXR activation by HIF-1 was responsible, at least partially, for the induction of MDR1.

The stability and activity of HIF-1 are tightly regulated, primarily through an oxygen-dependent pathway. Under normoxic conditions, HIF-1α is hydroxylated at conserved proline residues by prolyl hydroxylases and ubiquitinated by a pVHL-containing E3 ubiquitin ligase, resulting in rapid degradation by proteasomes. Under hypoxic conditions, HIF-1α is stabilized and regulates the expression of target genes^[23]^. HIF-1 also has been reported to be regulated by cytokines, growth factors, environmental stimulators, and other signaling molecules in an oxygen-independent manner^[24]^. We found that PXR binds to ectopically expressed HIF-1 under normoxic conditions and endogenous HIF-1 under hypoxic conditions. While the isoform (110 KDa) of HIF-1 was found to interact with PXR under normoxic conditions, it is the putative isoform HIF-1 (120 KDa) that interacts with PXR under hypoxic conditions. There are isoforms of HIF-1 that can suppress the activity and down-regulate mRNA expression of HIF-1^[25,26]^. Further studies are needed to determine whether and how PXR regulates the activities of HIF-1 during the tumor hypoxia response outside hypoxia-associated drug resistance.

In summary, our study identified a novel interaction between nuclear receptor PXR and transcriptional factor HIF-1. Under hypoxic conditions, HIF-1 increased the activity of PXR, leading to increased expression of MDR1 and increased resistance of prostate cancer cells to chemotherapy. PXR physically interacted with HIF-1 under hypoxic conditions. Our study highlights the pleiotropic effects of PXR on prostate cancer cells regarding the tumor hypoxia response and resistance to chemotherapy.

## DECLARATIONS

### Copyright

© The Author(s) 2023.
